# Caffeic Acid Phenethyl Ester Is a Potential Therapeutic Agent for Oral Cancer

**DOI:** 10.3390/ijms160510748

**Published:** 2015-05-12

**Authors:** Ying-Yu Kuo, Wai-Tim Jim, Liang-Cheng Su, Chi-Jung Chung, Ching-Yu Lin, Chieh Huo, Jen-Chih Tseng, Shih-Han Huang, Chih-Jen Lai, Bo-Chih Chen, Bi-Juan Wang, Tzu-Min Chan, Hui-Ping Lin, Wun-Shaing Wayne Chang, Chuang-Rung Chang, Chih-Pin Chuu

**Affiliations:** 1 Institute of Cellular and System Medicine, National Health Research Institutes, Miaoli 35053, Taiwan; E-Mails: 034279@nhri.org.tw (Y.-Y.K.); liangcheng610@nhri.org.tw (L.-C.S.); cylin071@gmail.com (C.-Y.L.); s103080810@m103.nthu.edu.tw (C.H.); s100080826@m100.nthu.edu.tw (J.-C.T.); hshf7855@gmail.com (S.-H.H.); krad0127@gmail.com (C.-J.L.); s102080545@m102.nthu.edu.tw (B.-C.C.); bijuanwang1004@gmail.com (B.-J.W.); 2 Institute of Biotechnology, National Tsing Hua University, Hsinchu City 30013, Taiwan; E-Mail: crchang@life.nthu.edu.tw; 3 College of Life Science, National Tsing Hua University, Hsinchu City 30013, Taiwan; 4 Department of Pediatrics, Mackay Memorial Hospital, Taipei City 10449, Taiwan; E-Mail: waitim@ms1.mmh.org.tw; 5 Department of Medicine, Mackay Medical College, New Taipei City 25245, Taiwan; 6 Department of Early Childhood Care and Education, Mackay Junior College of Medicine, Nursing, and Management, New Taipei City 25245, Taiwan; 7 Department of Health Risk Management, China Medical University, Taichung City 40402, Taiwan; E-Mail: cjchung@mail.cmu.edu.tw; 8 Department of Life Sciences, National Central University, Taoyuan City 32001, Taiwan; 9 Institute of Molecular and Cellular Biology, National Tsing Hua University, Hsinchu City 30013, Taiwan; 10 Department of Medical Education and Research, China Medical University Beigang Hospital, Yunlin County 65152, Taiwan; E-Mail: ziv.j@yahoo.com.tw; 11 Department of Medical Education and Research, Tainan Municipal An-Nan Hospital, China Medical University, Tainan City 70965, Taiwan; 12 National Institute of Cancer Research, National Health Research Institutes, Miaoli 35053, Taiwan; E-Mails: diablofish@nhri.org.tw (H.-P.L.); wayne@nhri.org.tw (W.-S.W.C.); 13 Graduate Institute of Basic Medical Science and PhD Program for Aging, China Medical University, Taichung City 40402, Taiwan; 14 Biotechnology Center, National Chung Hsing University, Taichung City 40227, Taiwan; 15 PhD Program in Environmental and Occupational Medicine, Kaohsiung Medical University, Kaohsiung City 80708, Taiwan

**Keywords:** oral cancer, caffeic acid phenethyl ester, Akt, MMP, NF-κB, cell proliferation, cell cycle arrest, apoptosis, metastasis

## Abstract

Head and neck cancers, which affect 650,000 people and cause 350,000 deaths per year, is the sixth leading cancer by cancer incidence and eighth by cancer-related death worldwide. Oral cancer is the most common type of head and neck cancer. More than 90% of oral cancers are oral and oropharyngeal squamous cell carcinoma (OSCC). The overall five-year survival rate of OSCC patients is approximately 63%, which is due to the low response rate to current therapeutic drugs. In this review we discuss the possibility of using caffeic acid phenethyl ester (CAPE) as an alternative treatment for oral cancer. CAPE is a strong antioxidant extracted from honeybee hive propolis. Recent studies indicate that CAPE treatment can effectively suppress the proliferation, survival, and metastasis of oral cancer cells. CAPE treatment inhibits Akt signaling, cell cycle regulatory proteins, NF-κB function, as well as activity of matrix metalloproteinase (MMPs), epidermal growth factor receptor (EGFR), and Cyclooxygenase-2 (COX-2). Therefore, CAPE treatment induces cell cycle arrest and apoptosis in oral cancer cells. According to the evidence that aberrations in the EGFR/phosphoinositide 3-kinase (PI3K)/protein kinase B (Akt) signaling, NF-κB function, COX-2 activity, and MMPs activity are frequently found in oral cancers, and that the phosphorylation of Akt, EGFR, and COX-2 correlates to oral cancer patient survival and clinical progression, we believe that CAPE treatment will be useful for treatment of advanced oral cancer patients.

## 1. Introduction

Head and neck cancers include cancers evolved from the oral cavity, pharynx, larynx, paranasal sinuses and nasal cavity, and salivary glands. Head and neck cancers rank as the sixth most common cancer worldwide, affecting 650,000 people and causing 350,000 deaths per year [1,2]. Oral cancer is the most common type of head and neck cancer and caused 135,000 deaths worldwide in 2013 [3]. According to the Surveillance, Epidemiology, and End Results Program (SEER), the five-year survival rate of oral cavity and pharynx cancer in the United States is 63%. The poor prognosis of oral and oropharyngeal squamous cell carcinoma (OSCC), which account for 90% of the oral cancers, is due to the low response rate to current therapeutic drugs [2,4].

Propolis (bee glue) is produced by honeybees through mixing the secretions of their hypopharyngeal glands with the digested product of resins collected from leaves, flowers, and tree barks, which is used to build honeybee hives [5]. Propolis protects honey bee hives against rain and is a very sticky substance that prevents insects, rodents, and robber bees from entering the hives [5]. Propolis also acts as a biocide to kill invasive bacteria, fungi, or even larvae [5]. Propolis is a natural medicine used for hundreds of years and is being sold as dietary supplements. Propolis has been reported to exhibit anti-bacterial, anti-viral, fungicidal, anti-oxidative, free radical scavenging, immuno-modulatory, and anti-cancer activities [5]. Clinical trials, animal models, and cell culture experiments indicated that treatment with propolis is beneficial in dental application [5,6], such as decreasing the dentinal hypersensitivity [7,8], defending the dental caries [6], decreasing the oral mucositis resulted from chemotherapy [9,10,11], fortifying the salivary gland function [12], reducing the xerostomia due to radiotherapy to salivary glands [12], preventing oral cancer [13], inhibiting plaque and having anti-inflammatory effects [14], increasing the periodontal ligament cell viability of avulsed teeth [15], stimulating wound healing in the dental pulp [16], acting as an analgesic [17], and as an antibacterial agent against oral pathogens [18,19,20], declining the quantity of Enterococcus faecalis in root canals [21], lessening gingivitis [22], reducing recurrent aphthous stomatitis (RAS) [23,24], protecting the oral mucosa [25], and promoting wound healing after surgeries in the oral cavity [14]. Propolis is a complex mixture of more than 300 different natural constituents including phenolic acid, terpenes, cinnamic acid, caffeic acid, aromatic aldehydes, alcohols, amino acids, fatty acids, vitamins (A, B1, B2, B3, and B7), esters, minerals, essential oils, and flavonoids (flavones, flavonols, and flavanones) [5]. The component of propolis varies according to the species of bees, the difference of geological region, and the different kinds of plants and flowers the bees collect, which makes it difficult to define the actual molecular mechanisms of the anti-cancer activity of propolis.

**Figure 1:**
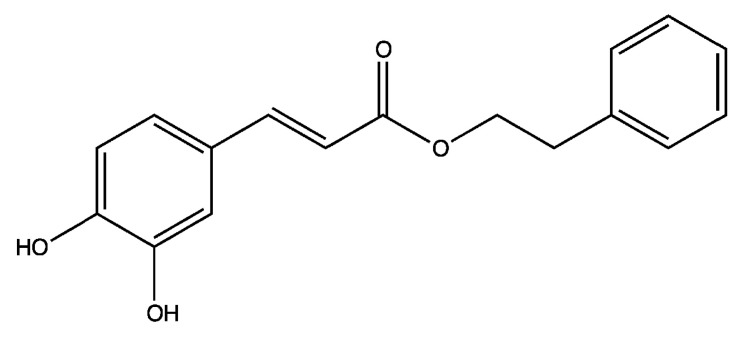
Structure of caffeic acid phenethyl ester (CAPE).

Caffeic acid phenethyl ester (CAPE) (Figure 1) is one of the most bioactive components extracted from honeybee hive propolis [26,27]. CAPE treatment exhibits anti-carcinogenic, anti-inflammatory, anti-viral, and immuno-modulatory properties [28]. Several recent studies indicate that CAPE treatment suppresses proliferation, survival, and invasion of human oral cancer cells. We therefore discuss the potential of using CAPE as a treatment for patients with oral cancer in this review article.

## 2. Oral Cancer

There are several types of oral cancers. The majority (>90%) of oral cancers are OSCC [2,4,29]. OSCCs arise in the oral cavity, oropharynx, larynx or hypopharynx and are characterized by poor prognosis and low survival rate [30]. The incidence and mortality rates worldwide of OSCC are approximately 5.9 and 3.3 per 100,000 persons per year, respectively [31]. Forty thousand OSCC cases were diagnosed and 8000 patients died from OSCC in the United States in 2012 [29,32]. The incidence of oral cancer is highest in Eastern and Southern Asia as well as central African countries and oral cancer accounts for 40%–50% of all malignancies in South and South-East Asian countries [33,34]. Environmental carcinogens, such as betel quid chewing, tobacco smoking, and alcohol drinking, have been identified as major risk factors for head and neck cancers [35]. According to the statistics of Taiwanese Department of Health, oral cancer ranks as the fourth most common cancer and the fifth leading cause of cancer death in Taiwanese males in 2012. The majority of oral cancer patients in Taiwan are regular users of betel quid [35]. Betal quid is a combination of betel leaf, areca nut, and slaked lime [35]. The cumulative effect of betel quid chewing, alcohol drinking, and tobacco smoking increases the risk of oral cancer up to 123-fold in Taiwanese patients [35].

## 3. Caffeic Acid Phenethyl Ester (CAPE) and Anticancer Effects

Caffeic acid phenethyl ester (CAPE) (Figure 1) is one of the main bioactive components extracted from honeybee hive propolis. CAPE is a strong antioxidant [26,27] and a lipophilic derivative of caffeic acid and a phenolic antioxidant structurally related to 3,4-dihydroxycinnamic acid. CAPE is a well-known NF-κB inhibitor [27]. CAPE treatment (50–80 μM) inhibits the activation of NF-κB via preventing the translocation of the p65 unit of NF-κB [27] and blocking the binding between NF-κB and DNA [27]. CAPE is an excellent anti-cancer agent. Treatment with CAPE inhibits the transformation of normal cells to cancer cells [36] as well as suppressing the proliferation of several human cancer cell lines, such as breast [37,38], prostate [39,40,41,42], lung [43,44], head and neck [45], cholangio [46], and cervical [47] cancer cells. Non-cancer human cells are much more resistant to CAPE treatment, indicating the potential selective cytotoxic effect against cancer cells of CAPE treatment [39,43,45,48]. CAPE treatment induces apoptosis or cell cycle arrest (G1 or G2/M) in different types of cancer cells [36,38,39,43,47,49,50,51,52,53,54,55,56,57]. CAPE treatment suppresses cancer cell movement and migration [58,59]. Oral administration or intraperitoneal (i.p.) injection of CAPE prevents cancer initiation, tumor growth, and cancer metastasis of colon, liver, and breast cancers [46,60,61,62,63,64,65,66,67,68] in animal models. CAPE is distributed extensively into animal tissues and is eliminated rapidly with a short half-life [69]. Intraperitoneal injection of CAPE at 10–30 mg/kg for seven days does not show toxic effects or affect the body weight of mice [65]. Additionally, CAPE treatment inhibits the proliferation of breast cancer stem cells [70]. CAPE treatments have also been shown to sensitize cancer cells to chemotherapeutic drugs and radiation treatment by inhibiting pathways that lead to treatment resistance as well as protecting important organs under chemotherapy and radiation treatments in animal models [71,72,73,74,75,76,77,78,79,80,81]. As CAPE exhibits very little or no toxic side effects, it is a potentially good candidate as a cancer therapeutic agent. Treatment with CAPE not only may suppress tumor growth in patients but also may protect patients from chemotherapy or radiation therapy.

## 4. Chemoprevention Effects of CAPE on Oral Cancer Cells

The epidermal growth factor receptor (EGFR), a receptor tyrosine kinase (RTK), is the cell-surface receptor for members of the epidermal growth factor (EGF) family. Elevated gene expression of EGFR has been reported to be associated with poor prognosis in OSCC [82,83]. Treatment with 5–30 μM CAPE dose-dependently suppresses the total abundance and phosphorylation of EGFR in breast cancer cells [37]. Cyclooxygenase (COX), including COX-1 and COX-2, is the enzyme responsible for the formation of the prostanoids. Cyclooxygenase-1 (COX-1) is present in most tissues, while Cyclooxygenase-2 (COX-2) is present at sites of inflammation. Both mRNA and protein level of COX-2 (also known as prostaglandin-endoperoxide synthase 2) are highly up-regulated in OSCC [84] and in high-risk premalignant oral lesions [85]. Up-regulation of COX-2 correlates to higher lymph node metastasis, faster cell proliferative activity, and worse survival rate in patients with oral carcinoma [86]. CAPE suppresses COX-2 activity with an IC_50_ of 6.3 nM in J774 macrophages [87]. Treatment with 35–70 μM of CAPE inhibits the activity and expression of COX-2 in human 1483 oral squamous carcinoma cells [88]. According to the facts that CAPE treatment is able to inhibit the activity and abundance of EGFR and COX-2, we believe that administration of CAPE can prevent and delay the development or progression of oral cancers.

## 5. Anticancer Activity Effects of CAPE on Oral Cancer Cells

TW2.6 is an OSCC cancer cell line established from the untreated primary squamous cell carcinoma of the buccal mucosa from a 48-year-old Taiwanese male patient with a betel quid chewing and tobacco smoking habit [4,89]. TW2.6 cells have morphological features of keratinocytes with a doubling time of 24 h [4,89]. CAPE treatment dose-dependently suppressed the cell proliferation and soft-agar colony formation of TW2.6 cells [4], confirming the anticancer activity of CAPE against oral cancer cells. CAPE treatment suppressed the survival and growth of several head and neck cancer cell lines. CAPE treatment suppressed the proliferation of oral submucosus fibroblast (OSF), neck metastasis of Gingiva carcinoma (GNM), tongue squamous cell carcinoma (TSCCa), oral squamous cell carcinoma (SAS) cell line, oral epidermoid carcinoma-Meng 1 (OEC-M1) cell line, and OSCC cell line TW2.6 [4,45,90] when being treated for as short as 24 h (Table 1). The SAS cell line is derived from a human squamous cell carcinoma developed from the primary lesion of a tongue carcinoma in a Japanese patient. SAS cell line has wild-type p53, high invasive potential, and high migration ability [45,91]. The OEC-M1 cell line is a human oral epidermoid carcinoma generated from the primary lesion of an oral carcinoma in a Taiwanese patient, which is a p53 mutant, resistant to retinoic acid treatment, and expresses smaller sized hypophosphorylated Rb proteins compared with normal cells [45,92]. Normal human oral fibroblast (NHOF) cells and buccal mucosal fibroblast (BF) cells were more resistant to CAPE treatment with an IC_50_ of 175 and 341 μM, respectively [45,90], (Table 1), indicating that CAPE exhibits selective suppressive effect on human oral cancer cells. The suppressive effect of CAPE accumulated over time. The IC_50_ of CAPE to suppress proliferation of TW2.6 cells was 72.1, 41.5, and 19.0 μM for 24, 48, and 96 h of treatment, respectively. Although the IC_50_ of CAPE seems to be relatively high as compared to other chemotherapy drugs, CAPE can be applied in the form of topical cream or mouth rinsing solution. We believe that daily oral administration of CAPE for a week or longer is possible to cause regression of oral cancer cells.

**Table 1:** IC_50_ of CAPE for causing growth inhibition in non-malignant and cancerous human oral cell lines.

Human Oral Cell Lines	IC_50_ (μM)	Reference
Oral and oropharyngeal squamous cell carcinoma (OSCC) cell line TW2.6	72.1	[4]
Oral submucosus fibroblast (OSF)	90.6	[91]
Neck metastasis of Gingiva carcinoma (GNM)	101.0	[91]
Tongue squamous cell carcinoma (TSCCa)	120.9	[91]
Oral squamous cell carcinoma (SAS)	129.7	[45]
Oral epidermoid carcinoma-Meng 1 (OEC-M1)	159.2	[45]
Normal human oral fibroblast (NHOF)	175.0	[45]
Buccal mucosal fibroblast (BF)	341.0	[91]

5-Fluorouracil (also known as 5-FU) is a chemotherapy agent widely used for treating advanced head and neck cancer. 5-fluorouracil suppresses cancer cells by misincorporating fluoronucleotides into RNA and DNA as well as by inhibiting the nucleotide synthetic enzyme thymidylate synthase [93]. However, common undesired side effects of 5-fluorouracil include diarrhea, nausea, vomiting, mouth sores, poor appetite, watery eyes, photophobia, taste changes, metallic taste in mouth during infusion, and low blood counts. Co-treating TW2.6 cells with CAPE and 5-fluorouracil exhibited additive cell proliferation inhibition [4]. As the 5-fluorouracil is usually given as a topical cream or solution for oral cancer patients to form a thin coating at skin lesions, CAPE can be added into the 5-fluorouracil cream or solution for oral cancer treatment. Co-treatment of CAPE with 5-fluorouracil may decrease the uncomfortable syndromes or undesired side effects for patients using 5-fluorouracil.

## 6. Molecular Mechanism of Anticancer Activity of CAPE in Oral Cancer Cells

The PIK3CA oncogene is located at chromosome 3q26. Chromosome locus 3q26 is frequently amplified in OSCCs [94,95,96,97] and is associated with advanced stages as well as invasive and metastatic OSCC phenotypes [98,99,100]. Relatively high frequency of PIK3CA mutations was found in stage IV OSCC, suggesting that PI3K/Akt signaling may be involved in disease progression of OSCCs [101]. OSCC is relatively sensitive to radiotherapy, however, activated PI3K/Akt signaling enhances radiotherapy resistance in OSCC patients [102]. These findings suggested that PI3K/Akt signaling is a potential therapeutic target in OSCC patients. There are three mammalian isoforms of this enzyme, Akt1, Akt2, and Akt3 [103,104]. Two phosphorylation sites on Akt, threonine 308 and serine 473, regulate activity of Akt. Phosphorylation of Thr308 on Akt is activated by PDK1 [105,106], while phosphorylation of Ser473 on Akt is activated by mTOR kinase [107,108]. Phosphorylation of these two sites elevates activity of Akt. Akt regulates the phosphorylation of Gsk-3β Ser9, which in turn regulates the activity of Gsk-3β activity [109] as well as the abundance of β-catenin, cyclin D1, and cyclin E [108,109,110]. Forkhead box O1 (FOXO1) protein is a transcription factor that plays important roles in regulation of gluconeogenesis and glycogenolysis by insulin signaling. FOXO3a is a well-known tumor suppressor [111]. Recent studies suggest that FOXO1 is a tumor suppressor as well [112]. Phosphorylation of FOXO1 or FOXO3a by Akt inhibits their activity resulting in translocation of these proteins out of the nucleus [113]. Treatment with CAPE decreased protein abundance of signaling proteins involved in the Akt signaling pathway, including Akt, Akt1, Akt2, Akt3, phospho-Akt Ser473, phospho-Akt Thr308, Gsk3β, FOXO1, FOXO3a, phospho-FOXO1 Thr24, and phospho-FoxO3a Thr32 in Tw2.6 cells [4]. Overexpression of Akt1 or Akt2 in TW2.6 cells rescued growth inhibition caused by CAPE treatment [4], confirming that Akt is one of the main targets of CAPE in oral cancer cells.

The tumor microenvironment is the environment in which the cancer cell exists. The status of the surrounding blood vessels, immune cells, fibroblasts, stroma cells, hypoxia, signaling molecules, and the extracellular matrix (ECM) determine the conditions of the tumor microenvironment. CAPE treatment reduces the secretion of vascular endothelial growth factor (VEGF) through the inhibition of the ROS, PI3K and HIF-1α signaling pathways in human retinal pigment epithelial cells under hypoxic conditions [114]. Cancer metastasis is the major cause of cancer related death and involves the degradation of the ECM [115], which is regulated by the matrix metalloproteinases (MMPs) [116]. MMPs gene are expressed in response to stimulation by many pro-inflammatory cytokines such as TNF-α, interleukin (IL)-1β and IL-6. The activity of MMPs is regulated by several types of inhibitors, among them, tissue inhibitor of metalloproteinases (TIMPs) are the most important ones [117,118,119]. The balance between MMPs and TIMPs is responsible for the control of degradation of ECM proteins. MMP-2 and MMP-9 are the principal enzymes involved in the degradation of ECM [120,121] and both of these proteins have been reported to be overexpressed in oral cancers [122]. SCC-9 is a tumorigenic keratinocyte cell line cultured from human squamous cell carcinomas. CAPE treatment attenuates SCC-9 oral cancer cell migration and invasion at non-cytotoxic concentrations (0–40 μM) [123]. Western blot and gelatin zymography analysis indicates that CAPE down-regulated protein expression and enzymatic activity of MMP-2 [123]. CAPE exerts its inhibitory effects on MMP-2 expression and activity by up-regulating TIMP-2 and potently decreases migration by reducing focal adhesion kinase (FAK) phosphorylation and the activation of its downstream signaling molecules p38 mitogen-activated protein kinases (p38 MAPK) and c-Jun *N*-terminal kinases (JNKs) [123]. These data indicate that CAPE could potentially be used as a chemo-agent to prevent oral cancer metastasis and that the anticancer activity of CAPE is at least partially via regulation of the tumor microenvironment.

Nuclear factor kappa-B (NF-κB) is an important cell-survival signaling protein and plays a key role in regulating the cellular response to stress and the immune response to infection [124]. Dysregulation of NF-κB has been linked to cancer, inflammation, and autoimmune diseases [124]. NF-κB is one of the most important transcription factors for MMP-9 production [117,125,126]. High expression levels of NF-κB p65 and inhibitor of NF-κB kinase subunit alpha (IKKα) were found to correlate to invasiveness, metastasis, and anti-apoptotic activity of OSCC [127]. Oral cancer is associated with a high degree of local invasiveness [123]. CAPE treatment has been reported to suppress the activation of NF-κB induced by tumor necrosis factor (TNF) or inflammatory agents in a dose- and time-dependent manner [27]. CAPE prevents the translocation of the p65 subunit of NF-κB to the nucleus and delays nuclear factor of κ light polypeptide gene enhancer in B-cells inhibitor, alpha (IκBα) re-synthesis [27]. The effect of CAPE on inhibition of NF-κB binding to the DNA is target-specific [27]. Fifty μM or higher dosage of CAPE is an effective inhibitor of NF-κB activation in TW2.6 cells [4]. CAPE treatment (50 or 100 μM) suppress both the total abundance and the phosphorylation of NF-κB on Serine 536 [4]. Phosphorylation of NF-κB p65 at S536 is required for TNF-α-induced NF-κB activation [128]. Therefore, administration of CAPE can be a potential treatment for primary and metastatic OSCC by blocking the NF-κB survival pathway.

CAPE treatment decreased G1 cell population, increased G2/M cell population, and induced apoptosis in TW2.6 cells [4]. Skp2 is a member of the F-box protein family, which is responsible for ubiquitination and down-regulation of p27^Kip1^ and other proteins [129,130]. Treatment with CAPE decreases protein abundance of Rb, phospho-Rb Ser807/811, Skp2, and cyclin D1, but increases cell cycle inhibitor p27^Kip^ [4]. Rb is a tumor suppressor protein and is mutated or suppressed in several types of cancers [131]. Reduction in phosphorylation of Rb restricts cell proliferation by inhibiting activity of E2F transcriptional factors [132]. As the abundance of Rb is also suppressed by CAPE treatment in TW2.6 cells, the loss of Rb function may trigger either p53-dependent or p53-independent apoptosis [133]. Cyclin D1 is a protein encoded by *CCND1* gene and forms a complex with cyclin-dependent kinase 4 (CDK4) or CDK6. These complexes are essential for cell cycle G1/S transition [134]. Cyclin D1 interacts with Rb, and the expression of the *CCND1* gene is positively regulated by Rb [134]. Decline of phosphorylation of FOXO1 and FOXO3a caused by CAPE treatment elevates their tumor suppressor activity. Down-regulation of the protein level of Akt, phospho-Akt Ser473, phospho-Akt Thr308, Gsk3β, Skp2, phospho-Rb Ser807/811, phospho-FOXO1 Thr24, phospho-FOXO3a Thr32, and cyclin D1 coupled with the accumulation of p27^Kip1^ protein likely contribute to the induction of G2/M cell cycle arrest and growth inhibition in TW2.6 oral cancer cells.

## 7. Conclusions

**Figure 2:**
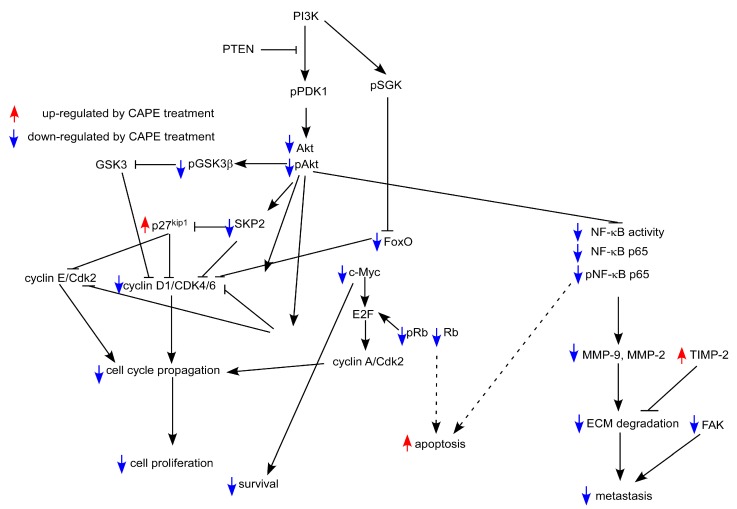
Putative model of anti-cancer effect of CAPE in human oral cancer cells. Protein abundance or activity being stimulated by CAPE treatment are labeled with red upward arrows, while those being suppressed by CAPE treatment are labeled with blue downward arrows. Arrows indicate activation of downstream signaling proteins, while bars mean inhibition of downstream signaling proteins. Dash lines indicated possible effects.

According to the above summaries in this review, there is strong evidence that CAPE treatment suppress proliferation, survival, metastasis, EGFR and COX-2 activity, PI3K-Akt signaling, and Skp2 in human oral cancer cells. We summarize the effects of CAPE treatment on different signaling proteins and the potential effect on cell survival, cell cycle, cell proliferation, and metastasis of oral cancer cells in Figure 2. The good bioavailability through the oral route, and the good historical safety profile of propolis, makes CAPE an ideal adjuvant agent for future oral cancer treatment. We believe that CAPE, being applied in the form of a topical cream or mouth rinsing solution, either alone or with 5-FU, can be a potentially effective treatment for patients with advanced oral cancer, targeting PI3K/Akt signaling, NF-κB, MMPs, and cell cycle regulatory proteins.
